# Tetra­kis(aceto­nitrile)copper(I) hydrogen oxalate–oxalic acid–aceto­nitrile (1/0.5/0.5)

**DOI:** 10.1107/S1600536813024914

**Published:** 2013-09-18

**Authors:** A. Timothy Royappa, Jacob R. Stepherson, Oliver D. Vu, Andrew D. Royappa, Charlotte L. Stern, Peter Müller

**Affiliations:** a Department of Chemistry, University of West Florida, 11000 University Parkway, Pensacola, FL 32514, USA; b Department of Chemistry, Northwestern University, 2145 Sheridan Road, Evanston, IL 60208, USA; c Department of Chemistry, Massachusetts Institute of Technology, 77 Massachusetts Avenue, Cambridge, MA 02139, USA

## Abstract

In the title compound, [Cu(CH_3_CN)_4_](C_2_HO_4_)·0.5C_2_H_2_O_4_·0.5CH_3_CN, the Cu^I^ ion is coordinated by the N atoms of four aceto­nitrile ligands in a slightly distorted tetra­hedral environment. The oxalic acid mol­ecule lies across an inversion center. The aceto­nitrile solvent mol­ecule is disordered across an inversion center and was refined with half occupancy. In the crystal, the hydrogen oxalate anions and oxalic acid mol­ecules are linked *via* O—H⋯O hydrogen bonds, forming chains along [010].

## Related literature

For background to tetra­kis­(aceto­nitrile)­copper(I) complexes, see: Morgan (1923 ▶); Heckel (1966 ▶); Kubas *et al.* (1979 ▶). For details of the affinity of nitrile ligands for Cu^I^ ions, see: Cotton *et al.* (1999 ▶). For the hard–soft acid–base theory, see: Pearson (1968 ▶). For the structure of the closely related tetra­kis­(aceto­nitrile)­copper(I) tetra­fluoro­borate, see: Jones & Crespo (1998 ▶).
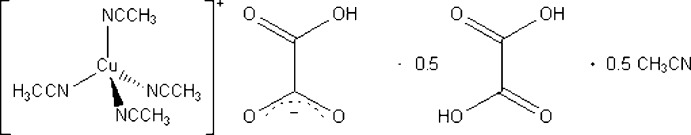


## Experimental

### 

#### Crystal data

[Cu(C_2_H_3_N)_4_](C_2_HO_4_)·0.5C_2_H_2_O_4_·0.5C_2_H_3_N
*M* *_r_* = 382.33
Monoclinic, 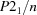

*a* = 9.5637 (4) Å
*b* = 5.5670 (2) Å
*c* = 32.0682 (12) Å
β = 92.901 (2)°
*V* = 1705.16 (11) Å^3^
*Z* = 4
Cu *K*α radiation
μ = 2.15 mm^−1^
*T* = 100 K
0.17 × 0.14 × 0.03 mm

#### Data collection

Bruker APEXII CCD diffractometer
Absorption correction: multi-scan (*SADABS*; Bruker, 2007 ▶) *T*_min_ = 0.715, *T*_max_ = 0.938
8349 measured reflections
2928 independent reflections
2745 reflections with *I* > 2σ(*I*)
*R*_int_ = 0.020

#### Refinement

*R*[*F* ^2^ > 2σ(*F* ^2^)] = 0.036
*wR*(*F* ^2^) = 0.098
*S* = 1.12
2928 reflections
236 parameters
18 restraints
H atoms treated by a mixture of independent and constrained refinement
Δρ_max_ = 0.89 e Å^−3^
Δρ_min_ = −0.50 e Å^−3^

### 

Data collection: *APEX2* (Bruker, 2007 ▶); cell refinement: *SAINT* (Bruker, 2007 ▶); data reduction: *SAINT*; program(s) used to solve structure: *SHELXS97* (Sheldrick, 2008 ▶); program(s) used to refine structure: *SHELXL97* (Sheldrick, 2008 ▶); molecular graphics: *OLEX2* (Dolomanov *et al.*, 2009 ▶); software used to prepare material for publication: *CHEMDRAW* (Cambridgesoft, 2003 ▶).

## Supplementary Material

Crystal structure: contains datablock(s) I. DOI: 10.1107/S1600536813024914/lh5649sup1.cif

Structure factors: contains datablock(s) I. DOI: 10.1107/S1600536813024914/lh5649Isup2.hkl

Additional supplementary materials:  crystallographic information; 3D view; checkCIF report

## Figures and Tables

**Table 1:** Hydrogen-bond geometry (Å, °)

*D*—H⋯*A*	*D*—H	H⋯*A*	*D*⋯*A*	*D*—H⋯*A*
O1—H1⋯O4^i^	0.85 (2)	1.69 (2)	2.538 (2)	176 (3)
O5—H5⋯O3	0.83 (2)	1.74 (2)	2.553 (2)	165 (4)

Symmetry code: (i) 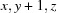.

**Fig. 1.**
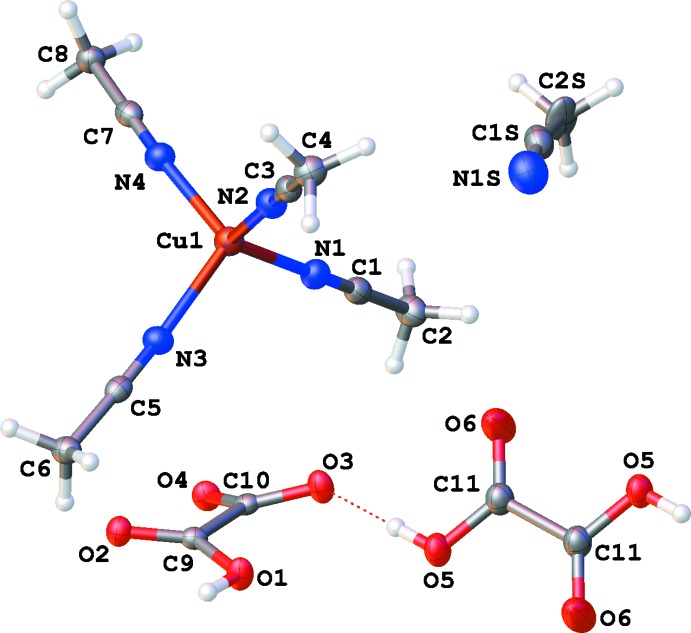
The molecular structure of the title compound with displacement ellipsoids shown at the 50% probability level. The symmetry complete oxalic acid molecule is shown and the acetonitrile solvent molecule is half occupancy. The dotted line indicates a hydrogen bond.

## supplementary crystallographic information

### 1. Comment

The tetrakis(acetonitrile)copper(I) ion, an important starting material for the synthesis of copper(I) complexes, was first synthesized as the nitrate salt (Morgan, 1923). Commonly available and easily synthesized compounds containing the [Cu(CH_3_CN)_4_]^+^ cation generally do contain weakly coordinating anions such as BF_4_^-^ (Heckel, 1966) or PF_6_^-^ (Kubas *et al.*, 1979). The structure of tetrakis(acetonitrile)copper(I) tetrafluoroborate already appears in the literature (Jones & Crespo, 1998).

In this work, we report the synthesis and crystal structure of a compound containing the [Cu(CH_3_CN)_4_]^+^ cation containing a potentially coordinating HC_2_O_4_^-^ anion, which does not coordinate to the metal center. This is in keeping with the known affinity of nitrile ligands for Cu^I^ (Cotton *et al.*, 1999), and also with the hard-soft acid-base theory (Pearson, 1968), which predicts a weak interaction between the "hard" hydrogen oxalate ligand and the "soft" Cu^+^ ion.

In the title compound, the [Cu(CH_3_CN)_4_]^+^ cation adopts a slightly distorted tetrahedral geometry. The HC_2_O_4_^-^ anion is not coordinated to the Cu^I^ center and the distances of the nearest O atoms to the Cu^I^ ion are all greater than 4.7 Å. The anion is non-planar, whereas the oxalic acid molecules are strictly planar, and reside on inversion centers. All the OH groups (in the hydrogen oxalate anion and in oxalic acid) in this structure are involved in intermolecular hydrogen bonding interactions with the carboxylate O atoms of the hydrogen oxalate anion, forming one-dimensional chains along [010]. The acetonitrile solvent molecules present in the structure are disordered, and positioned in linear channels between the [Cu(CH_3_CN)_4_]^+^ cations, parallel to the *b* axis.

### 2. Experimental

All manipulations were carried out under nitrogen. In a 10 ml round-bottom flask, 180 mg anhydrous oxalic acid (2 mmol), 0.143 g copper(I) oxide (1 mmol) and 7 ml degassed, dry acetonitrile were stirred together. All the red Cu_2_O powder dissolved in 2 min., forming a white precipitate and a clear, pale blue supernatant. After 15 min. of stirring, a copious amount of white and dark purple solids settled to the bottom of the flask. The dark purple solid was likely copper metal powder. This reaction mixture was stirred for 1 hr., then heated at 313K for 15 min., during which the white solid redissolved. Cooling to room temperature produced mm-sized, colorless, air- and moisture-sensitive, platelike crystals in 2 hrs.

### 3. Refinement

H atoms were placed in calculated positions with C—H = 0.96 Å and included in the refinement in a riding-motion approximation with *U*_iso_(H) = 1.5*U*_eq_(C). H atoms bonded to O atoms were refined independently with *U*_iso_(H) = 1.5*U*_eq_(O).

### Crystal data

**Table:**

[Cu(C_2_H_3_N)_4_](C_2_HO_4_)·0.5C_2_H_2_O_4_·0.5C_2_H_3_N	*F*(000) = 784
*M**_r_* = 382.33	*D*_x_ = 1.489 Mg m^−^^3^
Monoclinic, *P*2_1_/*n*	Melting point: not measured K
Hall symbol: -P 2yn	Cu *K*α radiation, λ = 1.54184 Å
*a* = 9.5637 (4) Å	θ = 2.8–66.4°
*b* = 5.5670 (2) Å	µ = 2.15 mm^−^^1^
*c* = 32.0682 (12) Å	*T* = 100 K
β = 92.901 (2)°	Plate, colorless
*V* = 1705.16 (11) Å^3^	0.17 × 0.14 × 0.03 mm
*Z* = 4	

### Data collection

**Table:**

Bruker APEXII CCD diffractometer	2928 independent reflections
Radiation source: fine-focus sealed tube	2745 reflections with *I* > 2σ(*I*)
Graphite monochromator	*R*_int_ = 0.020
φ and ω scans	θ_max_ = 66.5°, θ_min_ = 2.8°
Absorption correction: multi-scan (*SADABS*; Bruker, 2007)	*h* = −11→11
*T*_min_ = 0.715, *T*_max_ = 0.938	*k* = −4→6
8349 measured reflections	*l* = −37→36

### Refinement

**Table:**

Refinement on *F*^2^	Primary atom site location: structure-invariant direct methods
Least-squares matrix: full	Secondary atom site location: difference Fourier map
*R*[*F*^2^ > 2σ(*F*^2^)] = 0.036	Hydrogen site location: inferred from neighbouring sites
*wR*(*F*^2^) = 0.098	H atoms treated by a mixture of independent and constrained refinement
*S* = 1.12	*w* = 1/[σ^2^(*F*_o_^2^) + (0.0394*P*)^2^ + 2.6242*P*] where *P* = (*F*_o_^2^ + 2*F*_c_^2^)/3
2928 reflections	(Δ/σ)_max_ < 0.001
236 parameters	Δρ_max_ = 0.89 e Å^−^^3^
18 restraints	Δρ_min_ = −0.50 e Å^−^^3^

### Special details

**Table:**

Geometry. All e.s.d.'s (except the e.s.d. in the dihedral angle between two l.s. planes) are estimated using the full covariance matrix. The cell e.s.d.'s are taken into account individually in the estimation of e.s.d.'s in distances, angles and torsion angles; correlations between e.s.d.'s in cell parameters are only used when they are defined by crystal symmetry. An approximate (isotropic) treatment of cell e.s.d.'s is used for estimating e.s.d.'s involving l.s. planes.
Refinement. Refinement of *F*^2^ against ALL reflections. The weighted *R*-factor *wR* and goodness of fit *S* are based on *F*^2^, conventional *R*-factors *R* are based on *F*, with *F* set to zero for negative *F*^2^. The threshold expression of *F*^2^ > σ(*F*^2^) is used only for calculating *R*-factors(gt) *etc*. and is not relevant to the choice of reflections for refinement. *R*-factors based on *F*^2^ are statistically about twice as large as those based on *F*, and *R*- factors based on ALL data will be even larger.

### Fractional atomic coordinates and isotropic or equivalent isotropic displacement parameters (Å^2^)

**Table:**

	*x*	*y*	*z*	*U*_iso_*/*U*_eq_	Occ. (<1)
Cu1	0.00357 (4)	0.91194 (7)	0.139464 (11)	0.02214 (14)	
N1	0.1201 (2)	0.6945 (4)	0.10675 (7)	0.0249 (5)	
C1	0.1869 (3)	0.5512 (5)	0.09207 (8)	0.0223 (5)	
C2	0.2716 (3)	0.3648 (5)	0.07389 (8)	0.0270 (6)	
H2A	0.3673	0.3724	0.0863	0.040*	
H2B	0.2735	0.3897	0.0437	0.040*	
H2C	0.2312	0.2070	0.0794	0.040*	
N2	−0.1196 (2)	1.1336 (4)	0.10639 (7)	0.0246 (5)	
C3	−0.1829 (3)	1.2842 (5)	0.09054 (7)	0.0221 (5)	
C4	−0.2621 (3)	1.4787 (5)	0.07048 (8)	0.0261 (6)	
H4A	−0.2024	1.6211	0.0689	0.039*	
H4B	−0.3433	1.5164	0.0868	0.039*	
H4C	−0.2941	1.4297	0.0422	0.039*	
N3	0.1258 (2)	1.1393 (4)	0.17405 (7)	0.0233 (5)	
C5	0.1743 (2)	1.2988 (5)	0.19157 (7)	0.0197 (5)	
C6	0.2332 (3)	1.5037 (5)	0.21462 (8)	0.0228 (5)	
H6A	0.2187	1.6500	0.1979	0.034*	
H6B	0.3337	1.4783	0.2204	0.034*	
H6C	0.1868	1.5207	0.2410	0.034*	
N4	−0.0979 (2)	0.6916 (4)	0.17694 (7)	0.0236 (5)	
C7	−0.1385 (3)	0.5341 (5)	0.19555 (8)	0.0209 (5)	
C8	−0.1875 (3)	0.3283 (5)	0.21895 (8)	0.0227 (5)	
H8A	−0.1808	0.1826	0.2020	0.034*	
H8B	−0.2852	0.3540	0.2257	0.034*	
H8C	−0.1295	0.3102	0.2448	0.034*	
C9	0.5095 (2)	1.0524 (4)	0.17421 (7)	0.0159 (5)	
C10	0.5127 (2)	0.7966 (4)	0.15534 (7)	0.0163 (5)	
O1	0.53399 (19)	1.2210 (3)	0.14699 (5)	0.0218 (4)	
H1	0.533 (3)	1.355 (4)	0.1595 (9)	0.033*	
O2	0.48651 (18)	1.0852 (3)	0.21057 (5)	0.0203 (4)	
O3	0.48900 (19)	0.7711 (3)	0.11715 (5)	0.0230 (4)	
O4	0.53722 (18)	0.6316 (3)	0.18140 (5)	0.0203 (4)	
C11	0.4732 (3)	0.9454 (5)	0.02028 (8)	0.0263 (6)	
O5	0.5469 (2)	1.0158 (3)	0.05293 (5)	0.0268 (4)	
H5	0.519 (3)	0.957 (6)	0.0747 (7)	0.040*	
O6	0.3702 (3)	0.8228 (5)	0.02020 (6)	0.0575 (8)	
N1S	−0.002 (6)	0.4633 (17)	0.0000 (16)	0.0417 (18)	0.50
C1S	−0.0067 (6)	0.2602 (12)	0.00027 (18)	0.0306 (12)	0.50
C2S	0.011 (3)	0.016 (4)	−0.0032 (11)	0.042 (4)	0.50
H2S1	0.1114	−0.0221	−0.0015	0.064*	0.50
H2S2	−0.0304	−0.0391	−0.0301	0.064*	0.50
H2S3	−0.0347	−0.0653	0.0195	0.064*	0.50

### Atomic displacement parameters (Å^2^)

**Table:**

	*U*^11^	*U*^22^	*U*^33^	*U*^12^	*U*^13^	*U*^23^
Cu1	0.0241 (2)	0.0165 (2)	0.0258 (2)	0.00096 (15)	0.00143 (15)	−0.00010 (15)
N1	0.0270 (11)	0.0223 (11)	0.0256 (11)	−0.0008 (10)	0.0035 (9)	−0.0003 (9)
C1	0.0234 (13)	0.0219 (13)	0.0215 (12)	−0.0022 (11)	−0.0010 (10)	0.0013 (11)
C2	0.0266 (14)	0.0234 (14)	0.0308 (14)	0.0039 (11)	0.0001 (11)	−0.0054 (11)
N2	0.0261 (12)	0.0204 (11)	0.0271 (11)	0.0013 (10)	−0.0002 (9)	−0.0005 (9)
C3	0.0218 (12)	0.0233 (13)	0.0213 (12)	−0.0020 (11)	0.0017 (10)	−0.0042 (11)
C4	0.0273 (13)	0.0207 (13)	0.0305 (13)	0.0027 (11)	0.0019 (10)	0.0022 (11)
N3	0.0232 (11)	0.0201 (11)	0.0265 (11)	0.0000 (9)	−0.0004 (9)	0.0006 (10)
C5	0.0174 (12)	0.0210 (13)	0.0207 (12)	0.0041 (11)	0.0020 (9)	0.0044 (11)
C6	0.0211 (12)	0.0204 (13)	0.0269 (13)	0.0009 (10)	0.0014 (10)	−0.0028 (11)
N4	0.0238 (11)	0.0204 (11)	0.0267 (11)	−0.0003 (9)	0.0023 (9)	−0.0007 (10)
C7	0.0185 (12)	0.0203 (13)	0.0238 (12)	0.0022 (11)	0.0001 (9)	−0.0048 (11)
C8	0.0242 (13)	0.0199 (13)	0.0241 (12)	−0.0024 (11)	0.0012 (10)	−0.0002 (10)
C9	0.0142 (11)	0.0134 (11)	0.0198 (12)	0.0006 (9)	−0.0005 (9)	0.0016 (9)
C10	0.0131 (11)	0.0147 (12)	0.0215 (12)	0.0001 (9)	0.0027 (9)	0.0004 (10)
O1	0.0346 (10)	0.0102 (8)	0.0208 (8)	−0.0010 (8)	0.0037 (7)	0.0004 (7)
O2	0.0265 (9)	0.0156 (8)	0.0190 (9)	0.0014 (7)	0.0030 (7)	0.0000 (7)
O3	0.0353 (10)	0.0144 (8)	0.0191 (9)	−0.0016 (8)	−0.0005 (7)	−0.0009 (7)
O4	0.0279 (9)	0.0102 (8)	0.0227 (8)	0.0001 (7)	0.0002 (7)	0.0020 (7)
C11	0.0323 (15)	0.0246 (14)	0.0220 (13)	−0.0025 (12)	0.0013 (11)	0.0012 (11)
O5	0.0361 (11)	0.0260 (10)	0.0184 (9)	−0.0037 (8)	0.0004 (7)	0.0029 (8)
O6	0.0642 (16)	0.0815 (19)	0.0265 (11)	−0.0442 (15)	−0.0011 (10)	0.0054 (12)
N1S	0.048 (3)	0.033 (4)	0.043 (2)	0.009 (14)	−0.002 (2)	0.001 (13)
C1S	0.032 (3)	0.036 (3)	0.024 (2)	0.001 (3)	−0.002 (2)	−0.003 (3)
C2S	0.073 (8)	0.026 (5)	0.029 (8)	−0.019 (6)	0.017 (7)	0.007 (6)

### Geometric parameters (Å, º)

**Table:**

Cu1—N2	1.977 (2)	C7—C8	1.460 (4)
Cu1—N1	1.981 (2)	C8—H8A	0.9800
Cu1—N4	2.002 (2)	C8—H8B	0.9800
Cu1—N3	2.017 (2)	C8—H8C	0.9800
N1—C1	1.139 (3)	C9—O2	1.211 (3)
C1—C2	1.456 (4)	C9—O1	1.311 (3)
C2—H2A	0.9800	C9—C10	1.548 (3)
C2—H2B	0.9800	C10—O3	1.242 (3)
C2—H2C	0.9800	C10—O4	1.256 (3)
N2—C3	1.139 (3)	O1—H1	0.849 (18)
C3—C4	1.452 (4)	C11—O6	1.198 (4)
C4—H4A	0.9800	C11—O5	1.293 (3)
C4—H4B	0.9800	C11—C11^i^	1.547 (5)
C4—H4C	0.9800	O5—H5	0.829 (18)
N3—C5	1.137 (3)	N1S—C1S	1.132 (12)
C5—C6	1.457 (4)	C1S—C2S	1.38 (3)
C6—H6A	0.9800	C2S—H2S1	0.9800
C6—H6B	0.9800	C2S—H2S2	0.9800
C6—H6C	0.9800	C2S—H2S3	0.9800
N4—C7	1.140 (3)		
			
N2—Cu1—N1	115.66 (9)	H6A—C6—H6C	109.5
N2—Cu1—N4	114.26 (9)	H6B—C6—H6C	109.5
N1—Cu1—N4	104.25 (9)	C7—N4—Cu1	166.9 (2)
N2—Cu1—N3	102.50 (9)	N4—C7—C8	178.4 (3)
N1—Cu1—N3	110.40 (9)	C7—C8—H8A	109.5
N4—Cu1—N3	109.84 (9)	C7—C8—H8B	109.5
C1—N1—Cu1	171.6 (2)	H8A—C8—H8B	109.5
N1—C1—C2	178.9 (3)	C7—C8—H8C	109.5
C1—C2—H2A	109.5	H8A—C8—H8C	109.5
C1—C2—H2B	109.5	H8B—C8—H8C	109.5
H2A—C2—H2B	109.5	O2—C9—O1	125.5 (2)
C1—C2—H2C	109.5	O2—C9—C10	121.6 (2)
H2A—C2—H2C	109.5	O1—C9—C10	112.99 (19)
H2B—C2—H2C	109.5	O3—C10—O4	126.2 (2)
C3—N2—Cu1	171.1 (2)	O3—C10—C9	119.0 (2)
N2—C3—C4	179.2 (3)	O4—C10—C9	114.79 (19)
C3—C4—H4A	109.5	C9—O1—H1	108 (2)
C3—C4—H4B	109.5	O6—C11—O5	126.1 (2)
H4A—C4—H4B	109.5	O6—C11—C11^i^	122.0 (3)
C3—C4—H4C	109.5	O5—C11—C11^i^	111.8 (3)
H4A—C4—H4C	109.5	C11—O5—H5	112 (2)
H4B—C4—H4C	109.5	N1S—C1S—C2S	169 (3)
C5—N3—Cu1	166.5 (2)	C1S—C2S—H2S1	109.5
N3—C5—C6	178.5 (3)	C1S—C2S—H2S2	109.5
C5—C6—H6A	109.5	H2S1—C2S—H2S2	109.5
C5—C6—H6B	109.5	C1S—C2S—H2S3	109.5
H6A—C6—H6B	109.5	H2S1—C2S—H2S3	109.5
C5—C6—H6C	109.5	H2S2—C2S—H2S3	109.5

Symmetry code: (i) −*x*+1, −*y*+2, −*z*.

### Hydrogen-bond geometry (Å, º)

**Table:**

*D*—H···*A*	*D*—H	H···*A*	*D*···*A*	*D*—H···*A*
O1—H1···O4^ii^	0.85 (2)	1.69 (2)	2.538 (2)	176 (3)
O5—H5···O3	0.83 (2)	1.74 (2)	2.553 (2)	165 (4)

Symmetry code: (ii) *x*, *y*+1, *z*.
